# Natural History of Anal Papillomavirus Infection in HIV-Negative Men Who Have Sex With Men Based on a Markov Model: A 5-Year Prospective Cohort Study

**DOI:** 10.3389/fpubh.2022.891991

**Published:** 2022-05-11

**Authors:** Zewen Zhang, Xi Ling, Lirong Liu, Miaomiao Xi, Guozhen Zhang, Jianghong Dai

**Affiliations:** School of Public Health, Xinjiang Medical University, Urumqi, China

**Keywords:** human papillomavirus, incidence, clearance, HIV-negative, men who have sex with men, Markov model

## Abstract

**Objective:**

Men who have sex with men (MSM) are at increased risk for Human papillomavirus (HPV) infection compared to women and heterosexual men. We aimed to assess the incidence, clearance and duration of anal human papillomavirus (HPV) infection in HIV-negative MSM and the influencing factors in a 5-year prospective cohort study.

**Methods:**

From April 2016 to April 2021, HIV-negative MSM were recruited and followed every 6 months in Urumqi, Xinjiang, China. Questionnaires and anal swabs were collected at baseline and every 6 months. We detected 37 anal HPV genotypes using the HPV Geno Array Diagnostic Kit Test. Incidence and clearance rates of anal HPV infection and the influencing factors were estimated using a two-state Markov model.

**Results:**

A total of 585 MSM were included with a median age of 37 years [interquartile range (IQR): 31–43 years] and were followed for a median 2.8 years (IQR: 1.8–3.6 years). Incidence rates for any HPV and high-risk HPV (Hr-HPV) were 53.4 [95% confidence interval (CI): 49.1–58.0] and 39.0 (95% CI: 35.7–42.5)/1,000 person-months. Median duration of infection was 9.67 (95% CI: 8.67–10.86) and 8.51 (95% CI: 7.57–9.50) months, respectively. Clearance rates for any HPV and Hr-HPV were 50.9 (95% CI: 46.7–55.3) and 62.1 (95% CI: 56.8–66.7)/1,000 person-months, respectively. HPV16 and HPV6 had the highest incidence, lowest clearance rate and longest duration of infection among Hr-HPV and low-risk HPV (Lr-HPV) types, respectively. Receptive anal sex is a risk factor for any HPV [hazard ratio (HR) = 1.66, 95% CI: 1.16–2.38] and Hr-HPV infection (HR = 1.99, 95% CI:1.39–2.85). Recent anal sex without condom use was significantly associated with any HPV (HR = 1.80, 95% CI: 1.10–2.94) and Hr-HPV infection (HR = 2.60, 95% CI: 1.42–4.77). Age ≥35 years was significantly associated with Lr-HPV HPV infection only (HR = 1.40, 95% CI: 1.02–1.93). Both inserted and receptive anal sex (HR = 0.60, 95% CI: 0.40–0.89) and anal sex ≥2 times per week (HR = 0.61, 95% CI: 0.43–0.87) were associated with reduced Hr-HPV clearance. Six of the nine-valent vaccine types (HPV6, 11, 16, 18, 52 and 58) occurred most frequently, which indicates the need for high vaccination coverage in MSM.

**Conclusions:**

In this cohort study, high incidence and low clearance of any HPV, Hr-HPV and individual HPV infections emphasize the importance of MSM vaccination. Modifiable behavioral factors such as condoms and drug use should be incorporated into HPV prevention strategies.

## Introduction

Human papillomavirus (HPV) is one of the most common sexually transmitted infections (STIs) (1, 2). Men who have sex with men (MSM) are one of the most severely infected populations with HPV due to their specific and frequent sexual behavior (3–5). A meta-analysis showed that the prevalence of anal HPV infection in HIV-negative MSM was 64% (6), and may exceed 90% in those with HIV infection (7, 8). In China (9), the estimated prevalence of anal HPV among MSM was 85.1% (HIV-positive), 53.6% (HIV-negative), and 59.2% (unknown HIV status). Persistent infection with high-risk HPV (Hr-HPV) is a known cause of cervical, head and neck, penile and anal cancers (2, 10, 11), and low-risk HPV (Lr-HPV) can also cause genital warts (12, 13). MSM are about 20 times more likely to develop anal cancer than heterosexual men are (14, 15), and a recent study showed that HPV was detected in 88.3% of anal cancers (8).

Cervical HPV infection has been described in many studies (16–18) and there are many similarities between cervical and anal HPV infection (19, 20). For example, cervical squamous cell carcinoma (SCC) and anal SCC are both caused by persistent infection with Hr-HPV. It has been shown that there is a dramatic difference in the progression rate of cancer at the two sites (21, 22), suggesting that the natural history of anal and cervical HPV infection also differs significantly. Therefore, understanding and elucidating the severity and risk factors for HPV infection in MSM is necessary for prevention and vaccination in this population (23, 24). Unlike heterosexual men who may benefit in female-only vaccination settings, HPV-related disease is unlikely to decline in MSM populations (25). To the best of our knowledge, HPV infection among MSM in China is under-reported. Most of the studies were on the prevalence of HPV (26–29), and there has been an absence of long-term longitudinal studies. Longitudinal information on incidence, clearance and persistence of anal HPV infection among MSM in China is needed to further elucidate disease burden among MSM, in order to guide the implementation of prevention programs in this region.

Most previous studies have used traditional Cox proportional risk models (30, 31) and Poisson regression models (32, 33) to describe the natural history of HPV infection. These methods are commonly used to describe the risk of transition from one state to another and tend to focus only on the occurrence of the first outcome. However, infection with HPV clears within a certain period of time and re-infection may occur later, and Cox regression provides less information on the dynamic evolution of HPV infection. Apart from a few exceptions (34–36), most studies did not take into account the interval review nature of the data (i.e., the uncertainty of the exact timing of the event of interest). Multiple infections and clearances may have occurred between follow-up visits, but the assumption that the event occurred at the sampling date or at an intermediate point between visits would have biased estimates of incidence and clearance rates. Geskus et al. (35) used Markov models and Poisson regression to describe the natural history of Hr-HPV infection. They found that, compared with Markov models, Poisson regression showed that incidence estimates decreased by 22% for HPV16 and 53% for HPV56, and clearance decreased by 22% for HPV16 and 42% for HPV51.

We used a two-state Markov model to determine incidence, clearance and persistence of anal HPV infection in a longitudinal cohort of HIV-negative MSM in Urumqi, China over a 5-year period. Additionally, we assessed factors related to incidence and clearance of anal HPV in this cohort.

## Methods

### Enrollment and Follow-Up of Study Participants

**Figure 1:**
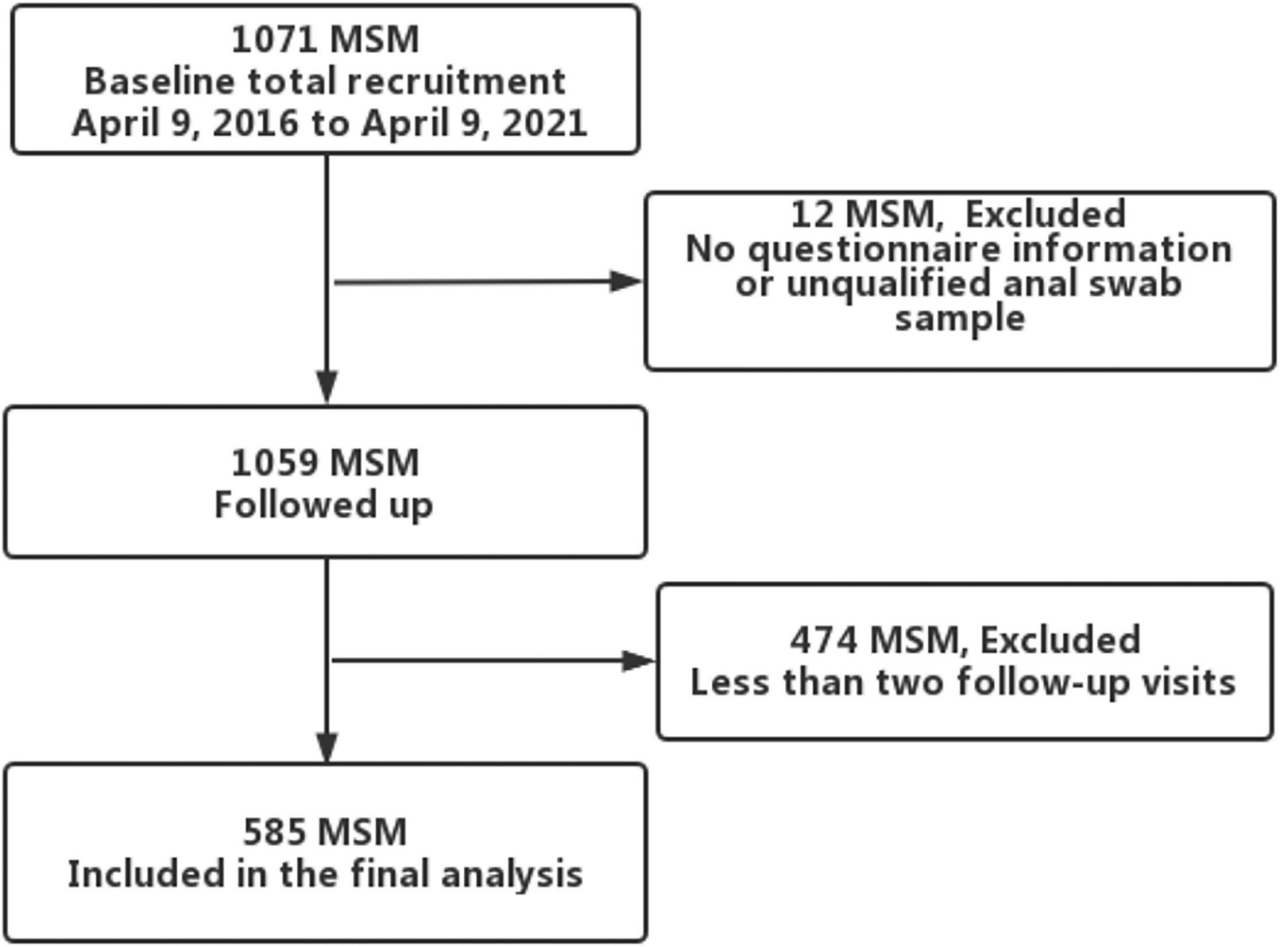
Flowchart for analytic dataset selection.

Since April 2016, we established an open prospective cohort of HPV among HIV-negative MSM in Urumqi, Xinjiang, China. The project recruited participants based on a local non-government organization (Xinjiang Dream Health Service Center, recognized by the health administration), through website advertising, WeChat and peer recruitment. Those who self-reported being at least 18 years old and having had homosexual sex at leasnce in the past 6 months were included. At baseline and at every 6-month study visit, participants completed a self-administered electronic questionnaire, including information on general socio-demographic and sexual behavior characteristics. A sample of 5 ml venous blood was collected for HIV screening. For the collection of anal samples, volunteers were trained by clinicians before the study; volunteers then instructed the participants to collect anal specimens during the study. Participants self-collected anal samples by rotating a swab inside the anal canal, and placed them in a 3-ml sample transport medium for HPV testing. Anal samples were stored at 4°C. Transported back to the laboratory for testing *via* a foam box with ice packs. Participants received 40 RMB (~$6.6) for transportation and time at baseline survey and every 6-month follow-up visit. Between April 9, 2016 and April 9, 2021, 1071 participants were recruited at baseline. Twelve participants were excluded due to missing baseline questionnaires or ineligible anal swab samples, and those with at least two follow-up visits and eligible samples were retained, resulting in 585 participants in the final analysis (Figure 1). All participants provided written informed consent. This study was reviewed and approved by the Ethics Committee of the First Affiliated Hospital of Xinjiang Medical University (Batch Number: 20160512-11).

### Sociodemographic and Sexual Behaviors Characteristics

Sociodemographic characteristics included the following variables: age, ethnicity, marital status, education, employment status, registered residence, earning per month, smoking status and alcohol consumption. Sexual behaviors included the following variables: age of sexual debut, self-reported sexual preference, homosexual anal sex in the past 6 months, anal sexual position, gender of sexual partners (past year or lifetime), number of sexual partners in the past 6 months, number of anal sex encounters in the last week, frequency of condom use during homosexual behavior in the past 6 months, drug use, self-reported STI in the past 6 months, circumcision history, and commercial sex with men in the past 6 weeks.

### HPV DNA Detection and Genotyping

Hybribio 37 HPV GenoArray Diagnostic Kit Test (Hybribio Biotech, Chaozhou, China) was use for HPV detection and genotyping, as described previously (27). The diagnostic kit is based on a flow-through hybridization and gene-chip method. The low-density gene chip was pre-fixed with 37 type-specific oligonucleotides and the genotype was analyzed using HybriMax (Hybribio Biotech). The final results were detected by colorimetric change on the membrane under direct visualization. Positive and negative controls were included in the GenoArray test kit in every polymerase chain reaction analysis, as well as during the hybridization process for quality control.

This method can simultaneously detect 37 HPV genotypes: HPV6, 11, 16, 18, 26, 31, 33–35, 39, 40, 42–45, 51–59, 61, 66–73, and 81–84.

### Statistical Analysis

All statistical analyses were conducted using SAS version 9.4 and R version 4.1.0. Continuous variables were described using medians and interquartile ranges (IQRs) if they had skewed distributions, and frequencies and percentages for categorical variables.

Incidence was defined as a positive detection of HPV type after HPV-negative test result of HPV type (0–1). Clearance was defined as a negative test result of an HPV type after positive detection of HPV type (1–0). The analysis included the following three groups of HPV: (i) any type: infection by at least one of the 37 detectable HPV types; (ii) Hr-HPV: infection by at least one high-risk type, i.e., HPV16, 18, 26, 31, 33, 35, 39, 45, 51–53, 56, 58, 59, 66–70, 73, 82; and (iii) Lr-HPV: infection by at least one low-risk type, i.e., HPV6, 11, 34, 40, 42–44, 54, 55, 57, 61, 71, 72, 81, 83, 84. Incidence and clearance rates, including 95% confidence intervals (CIs), were calculated based on the number of events divided by person-months of observations of event risk. Moreover, incidence and clearance rates along with duration of infection were also estimated for each HPV genotype.

We estimated incidence and clearance rates using a two-stage Markov model that allowed for interval censored event data (37). The two states were defined as HPV-DNA negative and HPV-DNA positive. Individuals could move from one state to another at any time point. Incidence was modeled as the rate of transition from a negative to a positive state; clearance as the rate of transition from a positive to a negative state. We allowed several infections and clearances to occur between two samples. The transition rate from one state to another was assumed to be constant over time, and the probability of transition between the states depended on the time between observations, and not the specific time of observation. We estimated bivariate and multifactorial hazard ratios (HRs) affecting infection and clearance transitions. The variables which showed a *p* < 0.05 in bivariate analyses were entered into the multivariate Markov model to estimate the HRs, as well as their 95% CIs. The Markov model was fitted in R4.1.0 using the MSM package.

## Results

### Participants Baseline Characteristics

From April 9, 2016 to April 9, 2021, a total of 585 MSM were enrolled and then contributed a total of 1595.4 person-years with a median follow-up time of 2.8 (IQR: 1.8–3.6) years. The median age of participants was 30 (IQR: 25–36) years, and 53.5% (*n* = 318) of the participants were in local households. Four hundred and fifty-six (79.5%) had a junior college degree or higher, and most of the participants (87.2%) were employed. Median age of sexual debut was 20 (18–23) years, and 453 (77.4%) identified themselves as homosexual. A total of 471 (80.5%) reported having had homosexual anal sex in the past 6 months and at baseline, and 330 (56.4%) reported always using a condom during anal sex with male partners in the preceding 6 months. Only 61 (10.4%) had more than five sexual partners and 173 (29.6%) had a history of drug use in the past 6 months. The socio-demographic and sexual behavioral baseline characteristics of the 585 MSM are reported in Table 1.

**Table 1:** Baseline demographics and sexual behavior characteristics of 585 MSM.

**Variable**	***n*** **(%)**
**Age (year)**	
Median (IQR)	30 (25–36)
**Age of sexual debut**	
Median (IQR)	20 (18–23)
**registered permanent residence**	
Urumqi, China	313 (53.5)
Other, China	272 (46.5)
**Ethnicity**	
Han	518 (88.6)
Other	67 (11.4)
**Educational Level**	
Less than or equal to high school	120 (20.5)
Higher professional education or university	465 (79.5)
**Employment**	
employed	510 (87.2)
Unemployment/Unemployed	75 (12.8)
**Marital Status**	
Unmarried	434 (74.2)
Married	106 (18.1)
Divorced/widowed	45 (7.7)
**Earnings per month**	
≤5,000 RMB	312 (53.3)
>5,000 RMB	273 (46.7)
**Sexual preference**	
Homosexuality	453 (77.4)
Heterosexuality/others	132 (22.6)
**Gender of Lifetime sexual partners**	
Male only	317 (54.2)
Both male and female	268 (45.8)
**Gender of sexual partners in the past year**	
Male only	459 (78.5)
Both male and female	126 (21.5)
**Homosexual anal sex in the past 6 months**	
YES	471 (80.5)
NO	114 (19.5)
**Anal sexual position**	
Mainly inserted anal sex	309 (52.8)
both inserted and receptive	27 (4.6)
Mainly receptive anal sex	249 (42.6)
**number of sexual partners past 6 months**	
<5	511 (87.4)
≥5	61 (10.4)
Missing	13 (2.2)
**Number of anal in the past a week**	
<2	448 (76.6)
≥2	124 (21.2)
Missing	13 (2.2)
**Condom use during latest anal sex**	
YES	448 (76.6)
NO	124 (21.2)
Missing	13 (2.2)
**Condom use during anal sex with male sex partners** **in the past 6 months**	
Always with condoms	330 (56.4)
Sometimes with condoms	199 (34.0)
Never with condoms	43 (7.4)
Missing	13 (2.2)
**Had commercial sex with men in the past 6 months**	
Yes	50 (8.6)
No	522 (89.2)
Missing	13 (2,2)
**Had sex with heterosexual partners in the past 6 months**	
Yes	94 (16.1)
No	491 (83.9)
**Circumcision history**	
Circumcised	360 (61.5)
Not circumcised	225 (38.5)
**Drug use past 6 months**	
Yes	173 (29.6)
No	412 (70.4)
**Smoking**	
Never smoked	339 (57.9)
Sometimes	87 (14.9)
Smoking every day	159 (27.2)
**Drinking**	
Never drink	177 (30.3)
Sometimes	392 (67.0)
Drink every day	16 (2.7)
**Number of sexual partners in last 6 months**	
Median (IQR)	2 (1–3)
Missing	13 (2.2)
**History of STI**	
No	531 (90.7)
Yes	54 (9.2)

At baseline, 50.3% (294/585) had any anal HPV infection. Hr-HPV was detected in 36.8% (215/585), while 26.3% (154/585) were positive for Lr-HPV (Supplemental Table 1). The self-reported prevalence of STI was 9.2%.

### Anal HPV Incidence and Clearance

Incidence and clearance rates for each HPV group are reported in Table 2. The incidence of any HPV infection was higher than the clearance rate, which was 53.8 (49.3–58.6)/1,000 person-months and 52.4 (48.0–57.0)/1,000 person-months, respectively. For the Hr-HPV and Lr-HPV groups, the incidence rate was lower than the clearance rate (IR/CR for Hr-HPV = 0.63, 95% CI: 0.55–0.71, IR/CR for Lr-HPV = 0.37, 95% CI:0.32–0.42). The estimated duration of any HPV and Hr-HPV was similar at 9.5 (95%CI: 8.5–10.7) and 8.4 (95% CI: 7.5–9.4) months, respectively. Lr-HPV had a duration of 6.5 (95%CI: 5.7–7.4) months.

**Table 2:** Incidence rate, clearance rate, incidence/clearance ratio, and duration of HPV infection in 585 MSM.

**Groups**	**Incidence rate^#^**	**Clearance rate^#^**	Ratio^*^	**Duration (months)**
Any-HPVs	53.4 (49.1,58.0)	50.9 (46.7,55.3)	1.05 (0.91,1.16)	9.67 (8.67,10.86)
Hr-HPVs	39.0(35.7,42.5)	62.1 (56.8,66.7)	0.63 (0.55,0.71)	8.51 (7.57,9.50)
Lr-HPVs	27.6 (25.1,20.4)	74.9 (67.9,82.3)	0.37 (0.32,0.42)	6.61 (5.84,7.44)

# *Incidence and clearance rates were calculated based on the number of events divided by person-months of observations of event risk, expressed in /1,000 person-months, 95% confidence intervals are in parentheses*.

* *Ratio of incidence and clearance rates*.

**Figure 2:**
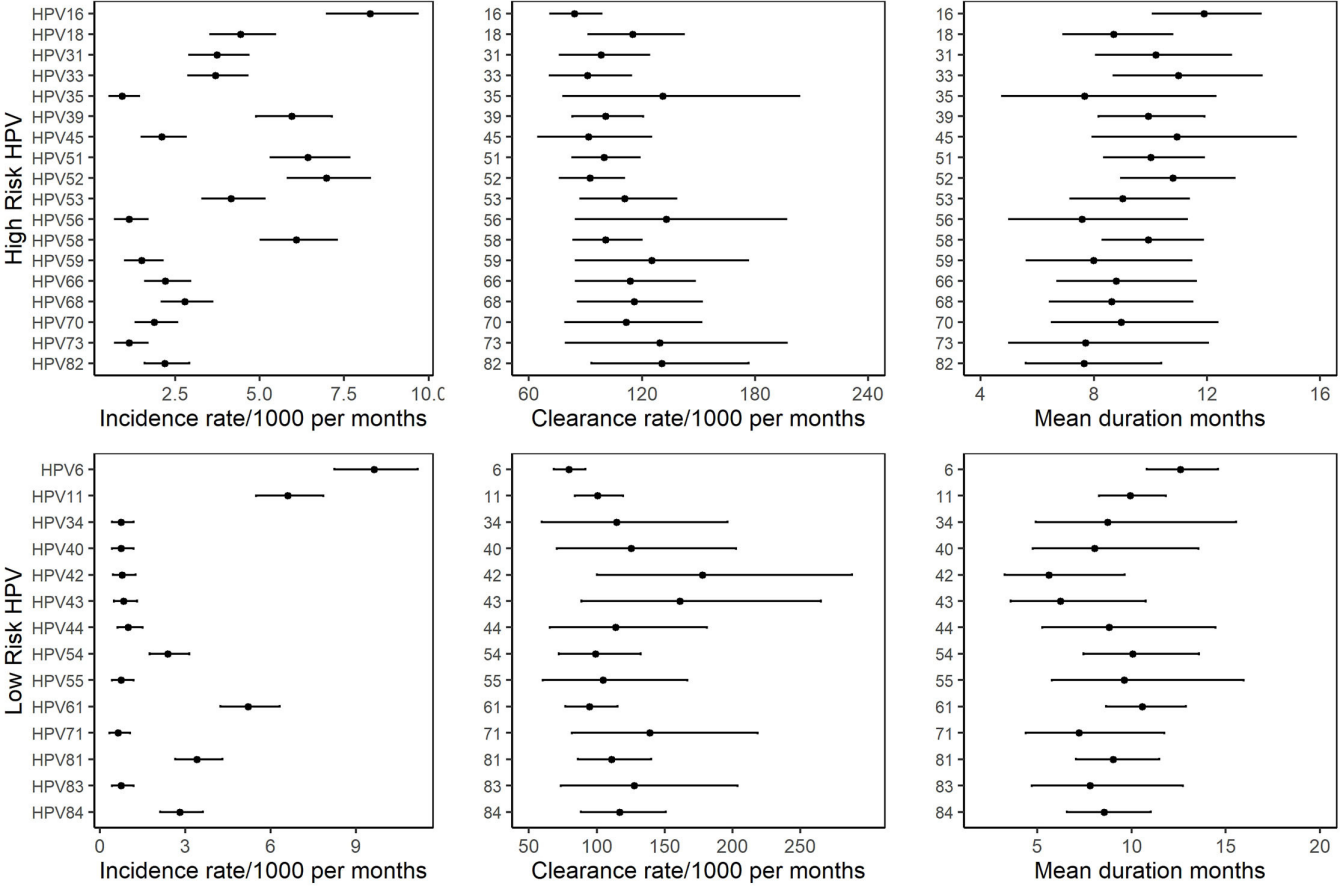
Incidence rates, clearance rates and duration of infection with 95% CI for individual HPV in 585 MSM based on the two-state Markov model.

The incidence rate of individual HPV infections varied substantially by HPV type. Among Hr-HPVs, the incidence rate ranged from 0.6 (95% CI: 0.6–1.4)/1,000 person-months for HPV69 to 8.3 (95%CI: 7.0–9.7)/1,000 person-months for HPV16. Among Lr-HPVs, the incidence rate ranged from 0.6 (95% CI: 0.3–1.0)/1,000 person-months for HPV71 to 9.6 (95% CI: 8.2–11.2)/1,000 person-months for HPV6 (Figure 2, Supplemental Table 2). The clearance rate of individual HPV infections also varied by HPV type. The clearance rate ranged from 84.1 (95% CI: 71.2–98.4)/1,000 person-months for HPV16 to 166.4 (95% CI: 79.9–299.9)/1,000 person-months for HPV69 among Hr-HPVs, and ranged from 79.5 (95% CI: 68.4–91.7)/1,000 person-months for HPV6 to 178.0 (95%CI: 100.3–288.2)/1,000 person-months for HPV42 among Lr-HPVs (Figure 2, Supplemental Table 2). The relatively high incidence and relatively low clearance of HPV6 and HPV16 make them the genotypes with the highest persistent infections, both when using clearance from prevalent-positive infections and incident-positive infections. Markov models showed that the duration of HPV6 and HPV16 infection was 12.6 (95% CI: 10.8–14.6) months and 11.9 (95% CI: 10.1–13.9) months, respectively (Figure 1, Supplemental Table 2). HPV42 and HPV56 had the shortest duration of infection in the low- and high-risk groups.

### Risk Factors for Incidence and Clearance of Anal HPV Infection

Multivariate analyses showed that MSM aged ≥35 years tended to be associated with a higher incidence of Lr-HPV (HR = 1.31, 95% CI: 1.02–1.93), while there was no significant association for any HPV and Hr-HPV (Tables 3, 4). Compared with inserted anal sex, receptive anal sex significantly increased the risk of infection with any HPV (HR = 1.66, 95% CI: 1.16–2.38) and Hr-HPV (HR = 1.99, 95% CI: 1.39–2.85). Increased incidence was observed only for infection with any HPV for both insertional and receptive anal sex (HR = 1.74, 95% CI: 1.06–2.84). Participants who did not use a condom during their latest anal sex showed a higher incidence of infection by any HPV (HR = 1.80, 95% CI: 1.10–2.94) and Hr-HPV (HR = 2.60, 95% CI: 1.42–4.77) compared to those that did use a condom. Drug use in the past 6 months (compared to no drug use), significantly increased the risk of infection with any HPV (HR = 1.42, 95% CI: 1.0–1.97) and Lr-HPV (HR = 2.18, 95% CI: 1.57–3.02), although the former was of borderline statistical significance. The incidence of infection with any HPV tended to increase in participants with a history of STI (HR = 2.17, 95% CI: 1.10–4.26), although not significantly, and a similar trend was observed for Hr-HPV.

**Table 3:** Bivariate and multivariate analyses of factors associated with incidence and clearance of Any HPV.

**Determinant**	**Incidence**		**Clearance**	
	**Bivariate**	**Multivariate**	**Bivariate**	**Multivariate**
**Any HPV type**	HR (95%CI)	HR (95%CI)	HR (95%CI)	HR (95%CI)
**Age (year)**				
≤34 years	ref		ref	
≥35 years	**1.33 (1.05,1.68)**	1.05 (0.71,1.55)	0.90 (0.71,1.15)	0.71 (0.48,1.07)
**Ethnicity**				
Han	ref		ref	
minority	**1.49 (1.02,2.18)**	0.86 (0.51,1.44)	0.76 (0.50,1.14)	0.46 (0.27,0.77)
**Marital status**				
Married	ref		ref	
Unmarried	**0.73 (0.55,0.98)**	0.70 (0.44,1.13)	1.09 (0.81,1.46)	0.87 (0.54,1.40)
Divorced/widowed	0.93 (0.60,1.45)	1.20 (0.62,2.32)	1.02 (0.65,1.61)	1.43 (0.74,2.75)
**Anal sexual position**				
Mainly inserted anal sex	ref		ref	
Both inserted and receptive	**1.64 (1.17,2.29)**	**1.74 (1.06,2.84)**	**0.93 (0.67,1.30)**	0.95 (0.59,1.52)
Mainly receptive anal sex	**1.37 (1.06,1.77)**	**1.66 (1.16,2.38)**	**0.65 (0.50,0.85)**	0.66 (0.46,0.95)
**Number of sexual partners past 6 months**				
<5	ref		ref	
≥5	**1.52 (1.00,2.30)**	1.28 (0.78,2.09)	1.19 (0.79,1.79)	1.07 (0.66,1.74)
**Latest anal sex condom use**				
Yes	ref		ref	
NO	**1.60 (1.11,2.31)**	**1.80 (1.10,2.94)**	1.21 (0.83,1.77)	1.47 (0.88,2.45)
**Circumcision**				
Yes	ref		ref	
No	0.88 (0.70,1.12)	0.91 (0.65,1.27)	**0.74 (0.58,0.94)**	0.74 (0.53,1.03)
**Drug use past 6 months**				
No	ref		ref	
Yes	**1.252 (0.98,1.61)**	**1.41 (1.00,1.97)**	1.09 (0.85,1.40)	1.15 (0.82,1.61)
**History of STI**				
No	ref		ref	
Yes	**1.71 (1.12,2.59)**	**2.17 (1.10,4.26)**	0.84 (0.55,1.30)	1.10 (0.54,2.22)

*Bold indicates P value < 0.05*.

**Table 4:** Bivariate and multivariate analyses of factors associated with incidence and clearance of Hr-HPVs and Lr-HPVs.

**Determinant**	**Incidence**		**Clearance**	
	**Bivariate**	**Multivariate**	**Bivariate**	**Multivariate**
**Hr- HPV type**	HR (95%CI)	HR (95%CI)	HR (95%CI)	HR (95%CI)
**Age**				
≤ 34 years	ref		ref	
≥35 years	**1.29 (1.02,1.64)**	1.14 (0.79,1.65)	0.95 (0.75,1.21)	0.88 (0.61,1.28)
**Marital status**				
Married	**ref**		ref	
Unmarried	**0.67 (0.50,0.91)**	0.68 (0.44,1.03)	0.94 (0.70,1.27)	0.90 (0.58,1.38)
Divorced/widowed	0.821 (0.53,1.27)	1.13 (0.57,2.25)	0.79 (0.51,1.24)	1.27 (0.63,2.52)
**Anal sexual position**				
Mainly inserted anal sex	ref		ref	
**Both inserted and receptive**	**1.39 (1.01,1.92)**	1.12 (0.73,1.71)	**0.71 (0.52,0.98)**	**0.60 (0.40,0.89)**
Mainly receptive anal sex	**1.57 (1.21,2.04)**	**1.99 (1.39,2.85)**	**0.68 (0.52,0.89)**	0.71 (0.50,1.02)
**Latest anal sex condom use**				
Yes	ref		ref	
NO	**2.02 (1.38,2.96)**	**2.60 (1.42,4.77)**	**1.53 (1.03,2.28)**	**2.11 (1.12,3.98)**
**Number of anal sex in the past a week**				
<2	ref		ref	
≥2	0.79 (0.60,1.05)	0.81 (0.57,1.134)	**0.64 (0.48,0.87)**	**0.61 (0.43,0.87)**
**Smoking status**				
Never smoked	ref		ref	
Sometimes	0.71 (0.51,1.00)	0.98 (0.61,1.58)	0.86 (0.61,1.20)	1.23 (0.77,1.95)
Smoking every day	**0.72 (0.54,0.94)**	0.74 (0.51,1.05)	0.81 (0.62,1.07)	0.77 (0.54,1.10)
**History of STI**				
No	ref		ref	
Yes	**1.51 (1.02,2.22)**	1.38 (0.83,2.29)	0.77 (0.51,1.16)	0.79 (0.46,1.34)
**Lr- HPV type**				
**Age**				
≤34 years	ref		ref	
≥35 years	**1.31 (1.01,1.69)**	**1.40 (1.02,1.93)**	0.89 (0.68,1.15)	1.11 (0.81,1.52)
**Gender of sexual partners in the past year**				
Male only				
Both male and female	0.87 (0.63,1.18)	0.85 (0.58,1.25)	**0.57 (0.42,0.77)**	**0.56 (0.39,0.82)**
**Number of sexual partners past 6 months**				
<5				
≥5	**1.81 (1.14,2.88)**	1.45 (0.89,2.37)	1.29 (0.82,2.02)	1.25 (0.79,1.99)
**Drug use past 6 months**				
No	ref		ref	
Yes	**1.90 (1.43,2.53)**	**2.18 (1.57,3.02)**	**1.67 (1.26,2.20)**	**1.75 (1.28,2.41)**
**Smoking status**				
Never smoked	ref		ref	
Sometimes	0.95 (0.65,1.37)	0.71 (0.44,1.14)	1.09 (0.75,1.57)	1.25 (0.80,1.94)
Smoking every day	1.13 (0.82,1.56)	1.09 (0.72,1.65)	**1.52 (1.11,2.09)**	1.45 (0.96,2.17)
**Drinking status**				
Never drink	ref		ref	
Sometimes	**1.36 (1.05,1.77)**	1.23 (0.89,1.71)	**1.37 (1.06,1.78)**	1.18 (0.85,1.62)
Drink every day	0.511 (0.20,1.28)	0.37 (0.12,1.18)	1.07 (0.48,2.34)	0.68 (0.21,2.20)

*Bold indicates P value < 0.05*.

In the multivariate analysis of the clearance of any HPV, none of the variables were significantly associated with clearance. Participants with both inserted and receptive anal sex had 0.6 times the hazard of Hr-HPV infection clearance compared to those with inserted anal sex (HR = 0.60, 95% CI: 0.40–0.89). Anal sex ≥2 times per week was associated with increased Hr-HPV clearance (HR = 0.61, 95% CI: 0.43–0.87). Most recent anal sex without a condom increased clearance of infection of Hr-HPV. The clearance of Lr-HPV was significantly lower in MSM who had both male and female sexual partners in the past year (HR = 0.56, 95% CI: 0.39–0.82). MSM who had used drugs in the past 6 months had higher clearance of Lr-HPV (HR = 1.75, 95% CI: 1.28–2.41).

## Discussion

We used a two-state Markov model to comprehensively assess the incidence, clearance and duration of anal infection with HPV (type 37) in HIV-negative MSM, as well as the factors influencing infection incidence and clearance.

In our study, the incidence of anal infection with any type of HPV and Hr-HPV was higher than that in HIV-negative MSM in other studies (4, 38–40); none of which took into account the nature of the data interval deletions. However, one study (41) in HIV-negative MSM using pre-exposure prophylaxis (PREP) found that the prevalence (92%) and incidence (86.2/1,000 person-months) of anal HPV infection were similar to those in HIV-positive MSM, which were higher than in our study. Few studies have reported the incidence of Lr-HPV infection in HIV-negative MSM. Dona MG et al. (34) reported higher incidence of Hr-HPV and Lr-HPV than we did, and they used the same statistical analyses as us to describe the natural history of HPV infection in HIV-negative MSM. They found that the clearance rate of any HPV infection was much lower than the incidence, which may explain the higher HPV prevalence at baseline (73.5%). However, in our study, the incidence of any HPV infection was similar to the clearance rate, and the prevalence of infection at baseline is also relatively low (50.3%). It is important to note that previous studies on the natural history of HPV have varied in the populations included, the HPV genotype tests used and number of genotypes tested, and the definitions of incidence and clearance, in addition to differences in the statistical methods used, making comparisons between studies difficult.

For individual HPV type infections and clearance, the highest incidence of anal HPV16 and HPV52 infections was found among Hr- HPV types, followed by HPV51, HPV58, HPV39, and HPV18. Similar to other studies, incidence rate of anal Hr-HPV infection was highest for HPV16, HPV51 and HPV52 in HIV-infected (4, 35, 39, 42) or in HIV-negative (34, 36, 39, 40, 43) MSM. We found that the highest incidence of infection with Lr-HPV was for HPV6, even higher than most Hr-HPVs. A meta-analysis (44) in China showed an unusually high prevalence of both HPV6 and HPV11 in MSM, but with the exception of HPV6 and 11, the prevalence of other low-risk genotypes was similar in heterosexual men and MSM. There are some specific differences in the incidence and prevalence of some types in different regions, suggesting that genotype validation methods may not be a key factor in the variation in incidence or prevalence, and that other parameters may play a greater role.

HPV16 had the lowest clearance rate compared to other Hr-HPVs, which is consistent with previous studies (15, 34, 36, 45), Evy Yunihastuti et al. (39). studied HIV-positive and HIV-negative MSM in South-East Asia, and found that HPV16 had the lowest clearance rate in both groups. HPV6 also had the lowest clearance, which partly explains the unusually high prevalence of HPV6 in Chinese MSM (44). HPV6 and HPV16 are also the two genotypes with the longest duration of infection. HPVs with the longest duration of infection may also have the highest oncogenic potential. HPV16 was found to persist longer than other Hr-HPV types in MSM, women and heterosexual men, which is consistent with the high pathogenicity of HPV16 infection in both sexes (17, 36, 46). A meta-analysis (10) showed that HPV16 was the most carcinogenic HPV type in the anus in both men and women, and the only HPV type that was significantly more common in cancer than in normal cytology. Currently, in China, the HPV vaccine is only available for women, and MSM cannot benefit from it. In addition, HPV infection is more common in MSM than in heterosexual men. In this study, six of the HPV types (HPV6, 11, 16, 18, 52 and 58) had higher incidence and lower clearance rates, given that this six types are included in the 9-valent vaccine, therefore, it is suggesting that the implementation of a vaccine program in the MSM may be important in reducing the prevalence of HPV infection in this population.

There was no significant association between age and incidence in most studies (34, 36, 39). In our study, age was significantly associated with an increased incidence of Lr-HPV infection. For infection clearance, a study of HIV-positive MSM by Geskus et al. (35) found that age had a significant non-linear effect; clearance was highest below age 25 years and lowest around 30 years. We also found that clearance was lower (although not significantly) in older participants in the any-HPV and Hr-HPV groups. Giuliano et al. (47) described the association between age and HPV clearance in HIV-negative MSM penile samples and found that Hr-HPV clearance increased with age.

Regarding sexual behavioral factors, receptive anal sex was independently associated with incidence of anal infection by any HPV and Hr-HPV in our study, which is consistent with previous studies (4, 44). A study (38) of multiple sites of HPV infection in adolescent MSM and the probability of transmission between sites found that younger, sexually inexperienced MSM had a higher incidence of anal HPV infection, higher probability of transmission from penis to anus, and significantly higher probability of transmission from penis to anus of 50% per partner than from anus to penis per partner. We also found that failure to use a condom during recent anal sex increased the risk of HPV infection. Therefore, during sex, if the penetrator does not use a condom, the penetrated partner may have a greater risk of infection. We also observed that STIs and drug use increased the risk of infection with any HPV and Hr-HPV. Drug use, number of sexual partners and history of STI may be substitutes for high-risk behaviors that could lead to an increased risk of exposure to HPV (15, 38).

We found that the number of anal sex encounters and both inserted and receptive anal sex were associated with Hr-HPV clearance failure. Condomless anal sex is a risky sexual behavior and can increase exposure to HPV. Therefore, failure of HPV clearance may be associated with re-exposure or re-infection through condomless sexual contact. Several studies have also reported an association between condomless anal sex and increased occurrence of HPV infection and failure of clearance (15). Our study found that the relationship between condomless anal sex and the occurrence of HPV infection was consistent with other studies (36, 46); however, condomless anal sex accelerated HPV clearance. Marra et al. (36) also found that never using a condom during anal sex increased the risk of transition from infected to uninfected state. We suggest that this finding could have arisen from MSM in a steady, perhaps sexually exclusive, relationship in which no or limited new HPV exposure occurred.

Our study had several strengths. First, our participants were recruited primarily through the community and may be a better representation of the MSM population in the local community than those recruited on an outpatient basis. Second, large and long follow-up enabled us to obtain a more accurate and comprehensive estimate of the natural history of anal HPV infection. Finally, the Markov model allowed us to more appropriately describe the dynamic process of HPV infection. The study also has some limitations. First, we assumed that the Markov model had a constant transfer probability from one state to another, independent of the time of transfer between states. However, the transfer rate of HPV was unlikely to have remained constant over a long observation period. Second, as with most studies, we could not really distinguish between new infection initiation and clearance and reactivation/suppression due to viral latency and immunity. Finally, this is an observational study and the results of this study may therefore be subject to unobserved confounding factors.

## Conclusion

Our study provides new data to explore the natural history and risk factors of anal HPV infection in HIV-negative MSM. This study found that anal HPV infection is common in HIV-negative MSM population. Our finding also confirms the most oncogenic character of HPV16 and the unusually high prevalence of HPV6. This emphasize the need to implement immunization programs in HIV-negative MSM to reduce the burden of HPV infection and related disease in MSM.

In addition, condom use and frequency of anal sex which are modifiable behavioral factors, should be included in preventive interventions to reduce the incidence of HPV infection and related diseases.

## Data Availability Statement

The original contributions presented in the study are included in the article/Supplementary Material, further inquiries can be directed to the corresponding author/s

## Ethics Statement

The studies involving human participants were reviewed and approved by the Ethics Committee of the First Affiliated Hospital of Xinjiang Medical University. The patients/participants provided their written informed consent to participate in this study.

## Author Contributions

ZZ conceived the presented idea, extracted and analyzed the data, and wrote the original draft. XL, LL, and MX reviewed and edited the original version of the article. GZ reviewed, edited, and formatted the article for submission. JD conceptualized the main idea, provided resources in data extraction and financial assistance during the whole study, and supervised the whole manuscript. All authors contributed to the article and approved the submitted version.

## Funding

This study was funded by National Natural Science Foundation of China (81560539 and 81860590), the National Major Science and Technology Project (2018ZX10721102-005), and the Natural Science Foundation of Xinjiang Uygur Autonomous Region (2019D01C204).

## Conflict of Interest

The authors declare that the research was conducted in the absence of any commercial or financial relationships that could be construed as a potential conflict of interest.

## Publisher's Note

All claims expressed in this article are solely those of the authors and do not necessarily represent those of their affiliated organizations, or those of the publisher, the editors and the reviewers. Any product that may be evaluated in this article, or claim that may be made by its manufacturer, is not guaranteed or endorsed by the publisher.

## References

[1] Schiffman M Castle PE. Human papillomavirus: epidemiology and public health. Arch Pathol Lab Med. (2003) 127:930–4. 10.5858/2003-127-930-HPEAPH 12873163

[2] de Martel C Plummer M Vignat J Franceschi S. Worldwide burden of cancer attributable to Hpv by site, country and Hpv type. Int J Cancer Res. (2017) 141:664–70. 10.1002/ijc.30716 28369882 PMC5520228

[3] Jacobs RJ Kane MN Ownby RL. Condom use, disclosure, and risk for unprotected sex in Hiv-negative midlife and older men who have sex with men. Am J Mens Healt. (2013) 7:186–97. 10.1177/1557988312463417 23093078

[4] Hernandez AL Efird JT Holly EA Berry JM Jay N Palefsky JM. Incidence of and risk factors for type-specific anal human papillomavirus infection among Hiv-Positive Msm. AIDS. (2014) 28:1341–9. 10.1097/QAD.0000000000000254 24959962 PMC4551512

[5] Wei F Gaisa MM D'Souza G Xia N Giuliano AR Hawes SE. Epidemiology of anal human papillomavirus infection and high-grade squamous intraepithelial lesions in 29 900 men according to HIV status, sexuality, and age: a collaborative pooled analysis of 64 studies. Lancet HIV. (2021) 8:e531–e43. 10.1016/S2352-3018(21)00108-9 34339628 PMC8408042

[6] Machalek DA Poynten M Jin F Fairley CK Farnsworth A Garland SM. Anal human papillomavirus infection and associated neoplastic lesions in men who have sex with men: a systematic review and meta-analysis. Lancet Oncol. (2012) 13:487–500. 10.1016/S1470-2045(12)70080-3 22445259

[7] del Amo J González C Geskus RB Torres M Del Romero J Viciana P. What drives the number of high-risk human papillomavirus types in the anal canal in Hiv-positive men who have sex with men? J Infect Dis. (2013) 207:1235–41. 10.1093/infdis/jit028 23325914

[8] Alemany L Saunier M Alvarado-Cabrero I Quirós B Salmeron J Shin HR. Human papillomavirus DNA prevalence and type distribution in anal carcinomas worldwide. Int J Cancer. (2015) 136:98–107. 10.1002/ijc.28963 24817381 PMC4270372

[9] Zhou Y Lin YF Gao L Dai J Luo G Li L. Human papillomavirus prevalence among men who have sex with men in china: a systematic review and meta-analysis. Eur J Clin Microbiol Infect Dis. (2021) 40:1357–67. 10.1007/s10096-021-04229-y 33768442

[10] Lin C Franceschi S Clifford GM. Human papillomavirus types from infection to cancer in the anus, according to sex and HIV status: a systematic review and meta-analysis. Lancet Infect Dis. (2018) 18:198–206. 10.1016/S1473-3099(17)30653-9 29158102 PMC5805865

[11] Safaeian F Ghaemimood S El-Khatib Z Enayati S Mirkazemi R Reeder B. Burden of cervical cancer in the eastern mediterranean region during the years 2000 and 2017: retrospective data analysis of the global burden of disease study. JMIR Public Health Surveill. (2021) 7:e22160. 10.2196/22160 33978592 PMC8156112

[12] Fuchs W Wieland U Skaletz-Rorowski A Brockmeyer NH Swoboda J Kreuter A. The male screening study: prevalence of HPV-related genital and anal lesions in an urban cohort of HIV-positive men in Germany. J Eur Acad Dermatol Venereol. (2016) 30:995–1001. 10.1111/jdv.13539 26833895

[13] Cerejeira A Cunha S Coelho R Macedo G Barkoudah E Azevedo F. Perianal warts as a risk marker for anal high-risk-human papillomavirus (HPV) Detection and HPV-associated diseases. J Eur Acad Dermatol Venereol. (2020) 34:2613–9. 10.1111/jdv.16834 32713086

[14] Silverberg MJ Lau B Justice AC Engels E Gill MJ Goedert JJ. Risk of Anal Cancer in Hiv-Infected and Hiv-Uninfected Individuals in North America. Clin Infect Dis. (2012) 54:1026–34. 10.1093/cid/cir1012 22291097 PMC3297645

[15] Patel P Bush T Kojic EM Conley L Unger ER Darragh TM. Prevalence, incidence, and clearance of anal high-risk human papillomavirus infection among HIV-infected men in the sun study. J Infect Dis. (2018) 217:953–63. 10.1093/infdis/jix607 29211874

[16] Jia H Ding L Han Y Lyu Y Hao M Tian Z. Genotype-specific distribution and change of high-risk human papillomavirus infection and the association with cervical progression risk in women with normal pathology and abnormal cytology in a population-based cohort study in China. J Cancer. (2021) 12:4379–88. 10.7150/jca.57993 34093838 PMC8176416

[17] Rositch AF Koshiol J Hudgens MG Razzaghi H Backes DM Pimenta JM. Patterns of persistent genital human papillomavirus infection among women worldwide: a literature review and meta-analysis. Int J Cancer. (2013) 133:1271–85. 10.1002/ijc.27828 22961444 PMC3707974

[18] Taguchi A Hara K Tomio J Kawana K Tanaka T Baba S. Multistate Markov model to predict the prognosis of high-risk human papillomavirus-related cervical lesions. Cancers. (2020) 12:270. 10.3390/cancers12020270 31979115 PMC7072567

[19] Gosens KC Richel O Prins JM. Human papillomavirus as a cause of anal cancer and the role of screening. Curr Opin Infect Dis. (2017) 30:87–92. 10.1097/QCO.0000000000000337 27845952

[20] Wasserman P Rubin DS Turett G. Review: anal intraepithelial neoplasia in HIV-infected men who have sex with men: is screening and treatment justified? AIDS Patient Care STDS. (2017) 31:245–53. 10.1089/apc.2017.0063 28530494

[21] Poynten IM Jin F Roberts JM Templeton DJ Law C Cornall AM. The Natural History of Anal High-Grade Squamous Intraepithelial Lesions in Gay and Bisexual Men. Clin Infect Dis. (2021) 72:853–61. 10.1093/cid/ciaa166 32342984

[22] Riibe M Sørbye SW Simonsen GS Sundsfjord A Ekgren J Maltau JM. Risk of cervical intraepithelial neoplasia grade 3 or higher (Cin3+) among women with HPV-test in 1990-1992, a 30-year follow-up study. Infect Agent Cancer. (2021) 16:46. 10.1186/s13027-021-00386-z 34158090 PMC8220730

[23] Chow EPF Tabrizi SN Fairley CK Wigan R Machalek DA Regan DG. Prevalence of human papillomavirus in teenage heterosexual males following the implementation of female and male school-based vaccination in Australia: 2014-2017. Vaccine. (2019) 37:6907–14. 10.1016/j.vaccine.2019.09.052 31562001

[24] Pollock KG Wallace LA Wrigglesworth S McMaster D Steedman N. HPV vaccine uptake in men who have sex with men in Scotland. Vaccine. (2019) 37:5513–4. 10.1016/j.vaccine.2018.11.081 30545714

[25] Chow EP Read TR Wigan R Donovan B Chen MY Bradshaw CS. Ongoing decline in genital warts among young heterosexuals 7 years after the Australian human papillomavirus (HPV) vaccination programme. Sex Transm Infect. (2015) 91:214–9. 10.1136/sextrans-2014-051813 25305210

[26] Ren X Ke W Zheng H Yang L Huang S Qin X. Human papillomavirus positivity in the anal canal in HIV-infected and HIV-uninfected men who have anal sex with men in Guangzhou, China: implication for anal exams and early vaccination. Biomed Res Int. (2017) 2017:2641259. 10.1155/2017/2641259 28133605 PMC5241445

[27] Tian T Mijiti P Bingxue H Fadong Z Ainiwaer A Guoyao S. Prevalence and risk factors of anal human papillomavirus infection among HIV-negative men who have sex with men in Urumqi City of Xinjiang Uyghur Autonomous Region, China. PLoS ONE. (2017) 12:e0187928. 10.1371/journal.pone.0187928 29141014 PMC5687769

[28] Zhang X Yu J Li M Sun X Han Q Li M. Prevalence and related risk behaviors of HIV, Syphilis, and anal HPV infection among men who have sex with men from Beijing, China. AIDS Behav. (2013) 17:1129–36. 10.1007/s10461-011-0085-x 22076229

[29] Li Z Zhang H Li X Yang Y Xin H Li M. Anal human papillomavirus genotyping among HIV-positive men who have sex with men in Xi'an, China. PLoS ONE. (2015) 10:e0125120. 10.1371/journal.pone.0125120 25923768 PMC4414525

[30] Beachler DC Pinto LA Kemp TJ Nyitray AG Hildesheim A Viscidi R. An examination of HPV16 natural immunity in men who have sex with men (MSM) in the HPV in men (HIM) study. Cancer Epidemiol Biomarkers Prev. (2018) 27:496–502. 10.1158/1055-9965.EPI-17-0853 29475967 PMC5884716

[31] Clarke MA Cheung LC Lorey T Hare B Landy R Tokugawa D. 5-year prospective evaluation of cytology, human papillomavirus testing, and biomarkers for detection of anal precancer in human immunodeficiency virus-positive men who have sex with men. Clin Infect Dis. (2019) 69:631–8. 10.1093/cid/ciy970 30418518 PMC6669293

[32] Mooij SH van Santen DK Geskus RB van der Sande MA Coutinho RA Stolte IG. The effect of HIV infection on anal and penile human papillomavirus incidence and clearance: a cohort study among MSM. AIDS. (2016) 30:121–32. 10.1097/QAD.0000000000000909 26474302

[33] Nyitray AG Carvalho da Silva RJ Chang M Baggio ML Ingles DJ Abrahamsen M. Incidence, duration, persistence, and factors associated with high-risk anal human papillomavirus persistence among HIV-negative men who have sex with men: a multinational study. Clin Infect Dis. (2016) 62:1367–74. 10.1093/cid/ciw140 26962079 PMC4872291

[34] Donà M Vescio M Latini A Giglio A Moretto D Frasca M. Anal human papillomavirus in HIV-uninfected men who have sex with men: incidence and clearance rates, duration of infection, and risk factors. Clin Microbiol Infect. (2016) 22:1004.e1–e7. 10.1016/j.cmi.2016.08.011 27585942

[35] Geskus RB González C Torres M Del Romero J Viciana P Masiá M. Incidence and clearance of anal high-risk human papillomavirus in HIV-positive men who have sex with men: estimates and risk factors. AIDS. (2016) 30:37–44. 10.1097/QAD.0000000000000874 26355673 PMC4674141

[36] Marra E Kovaleva A Bruisten S Vermeulen W Boyd A Schim van der Loeff MF. Incidence and clearance of anal high-risk human papillomavirus infections and their determinants over 5 years among human immunodeficiency virus-negative men who have sex with men. Clin Infect Dis. (2019) 68:1556–65. 10.1093/cid/ciy738 30169621

[37] Jackson CH. Multi-State Models for Panel Data: The Msm Package for R. J Stat Softw. (2011) 1:28. 10.18637/jss.v038.i08

[38] Zou H Tabrizi SN Grulich AE Hocking JS Bradshaw CS Cornall AM. Site-specific human papillomavirus infection in adolescent men who have sex with men (hyper): an observational cohort study. Lancet Infect Dis. (2015) 15:65–73. 10.1016/S1473-3099(14)70994-6 25435055

[39] Yunihastuti E Teeratakulpisarn N Jeo W Nilasari H Rachmadi L Somia I. Incidence, clearance, persistence and factors related with high-risk anal HPV persistence in South-East Asian MSM and transgender women. AIDS. (2020) 34:1933–41. 10.1097/QAD.0000000000002654 32773478 PMC7541660

[40] Sudenga S Torres B Silva R Villa L Lazcano-Ponce E Abrahamsen M. Comparison of the natural history of genital HPV infection among men by country: Brazil, Mexico, and the United States. Cancer Epidemiol Biomarkers Prev. (2017) 26:1043–52. 10.1158/1055-9965.EPI-17-0040 28446543 PMC5556383

[41] Cotte L Veyer D Charreau I Péré H Cua E Carette D. Prevalence and incidence of human papillomavirus infection in men having sex with men enrolled in a pre-exposure prophylaxis study: a sub-study of the agence nationale de recherches sur le sida et les hépatites virales “intervention préventive de l'exposition aux risques avec et pour les hommes gays” trial. Clin Infect Dis. (2021) 72:41–9. 10.1093/cid/ciaa002 31907521

[42] Darwich L Cañadas M Videla S Coll J Molina-López R Sirera G. Prevalence, clearance, and incidence of human papillomavirus type-specific infection at the anal and penile site of HIV-Infected Men. Sex Transm Dis. (2013) 40:611–8. 10.1097/01.OLQ.0000430798.61475.08 23859907

[43] Glick S Feng Q Popov V Koutsky LA Golden MR. High rates of incident and prevalent anal human papillomavirus infection among young men who have sex with men. Infect Dis. (2014) 209:369–76. 10.1093/infdis/jit441 23956439 PMC3883166

[44] Ma X Wang Q Ong JJ Fairley CK Su S Peng P. Prevalence of human papillomavirus by geographical regions, sexual orientation and HIV status in China: a systematic review and meta-analysis. Sex Transm Infect. (2018) 94:434–42. 10.1136/sextrans-2017-053412 29794242

[45] Ong J Walker S Grulich A Hoy J Read T Bradshaw C. Incidence, clearance, and persistence of anal human papillomavirus in men who have sex with men living with human immunodeficiency virus: implications for human papillomavirus vaccination. Sex Transm Dis. (2019) 46:229–33. 10.1097/OLQ.0000000000000958 30870323

[46] Moreira E Giuliano A Palefsky J Flores C Goldstone S Ferris D. Incidence, clearance, and disease progression of genital human papillomavirus infection in heterosexual men. J Infect Dis. (2014) 210:192–9. 10.1093/infdis/jiu077 24495910

[47] Giuliano AR Lee JH Fulp W Villa LL Lazcano E Papenfuss MR. Incidence and clearance of genital human papillomavirus infection in men (HIM): a cohort study. Lancet. (2011) 377:932–40. 10.1016/S0140-6736(10)62342-2 21367446 PMC3231998

