# Supplementary material for: Natural History of Anal Papillomavirus Infection in HIV-Negative Men Who Have Sex With Men Based on a Markov Model: A 5-Year Prospective Cohort Study
Source: Front Public Health. 2022 May 11;10:891991. doi: 10.3389/fpubh.2022.891991 (PMC9130828; doi:10.3389/fpubh.2022.891991)
Supplement: Supplementary file 1 [file Table_1.DOCX]

Supplemental Table 1 Prevalence of any anal HPV, high-risk HPV, and low-risk HPV in 585 MSM at baseline

| Group | No. of cases | Total number | Prevalence(%) |
| --- | --- | --- | --- |
| Any HPV | 294 | 585 | 50.3 |
| High risk HPV | 215 | 585 | 36.8 |
| Low risk HPV | 154 | 585 | 26.3 |

Supplemental Table 2 Incidence rate,Clearance rate and duration of infection for the individual high risk and low risk HPV in the 585 MSM

| HPV type | Incidence rate  x1000p-m(95%CI) | Clearance rate  x1000p-m(95%CI) | duration months  (95%CI) |
| --- | --- | --- | --- |
| High risk |  |  |  |
| 16 | 8.3 (7.0,9.7 ) | 84.1 (71.2, 98.4) | 11.9 (10.1, 13.9) |
| 18 | 4.4 (3.5, 5.5) | 115.0 (91.5, 142.3) | 8.7 (6.9, 10.8) |
| 31 | 3.7 (2.9, 4.7) | 98.2 (76.4, 123.8) | 10.2 (8.1, 12.9) |
| 33 | 3.7 (2.9, 4.6) | 91.0 (71.2, 114.2) | 11.0 (8.7, 13.9) |
| 35 | 0.9 (0.6, 1.4) | 130.9 (78.1, 203.4) | 7.7 (4.8, 12.3) |
| 39 | 6.0 (4.9, 7.1) | 100.7 (83.1, 120.7) | 9.9 (8.2, 11.9) |
| 45 | 2.1 (1.5, 2.8) | 91.7 (64.9, 125.0) | 10.9 (8.0, 15.1) |
| 51 | 6.4 (5.3, 7.7) | 99.9 (83.1, 118.8) | 10.0 (8.4, 11.9) |
| 52 | 7.0 (5.8, 8.3) | 92.4 (76.4, 110.6) | 10.8 (9.0, 13.0) |
| 53 | 4.2 (3.3, 5.1) | 110.9 (87.4, 138.3) | 9.0 (7.2, 11.4) |
| 56 | 1.1 (0.7, 1.7) | 132.9 (84.8, 196.5) | 7.6 (5.0, 11.3) |
| 58 | 6.1 (5.0, 7.3) | 100.7 (83.7, 119.9) | 9.9 (8.3, 11.9) |
| 59 | 1.5 (1.0, 2.1) | 125.1 (84.9, 176.3) | 8.0 (5.6, 11.4) |
| 66 | 2.2 (1.6, 3.0) | 113.7 (85.0, 148.2) | 8.8 (6.7, 11.6) |
| 67 | 0.5 (0.3, 0.9) | 157.1 (81.6, 269.0) | 6.4 (3.5, 11.4) |
| 68 | 2.8 (2.1, 3.6) | 115.8 (86.1, 151.8) | 8.6 (6.4, 11.5) |
| 69 | 0.6 (0.3, 1.0) | 166.4 (79.9, 299.9) | 6.0 (3.1, 11.7) |
| 70 | 1.9 (1.3, 2.6) | 111.7 (79.5, 151.7) | 9.0 (6.5, 12.4) |
| 73 | 1.1 (0.7, 1.7) | 129.5 (79.7, 196.8) | 7.7 (5.0, 12.0) |
| 82 | 2.2 (1.6,2.9) | 130.5 (93.3, 176.5) | 7.7 (5.6, 10.4) |
| Low risk |  | | |
| 6 | 9.6 (8.2, 11.2) | 79.5 (68.4, 91.7) | 12.6 (10.8, 14.6) |
| 11 | 6.6 (5.5, 7.9) | 100.7 (84.0, 119.5) | 9.9 (8.3, 11.8) |
| 34 | 0.7 (0.4, 1.2) | 114.6 (59.5, 196.3) | 8.7 (4.9, 15.5) |
| 40 | 0.7 (0.4, 1.2) | 125.3 (70.6, 202.8) | 8.0 (4.8, 13.5) |
| 42 | 0.8 (0.4, 1.2) | 178.0(100.3,288.2) | 5.6 (3.3, 9.6) |
| 43 | 0.8 (0.5, 1.3) | 161.2 (88.6, 265.3) | 6.2 (3.6, 10.8) |
| 44 | 1.0 (0.6, 1.5) | 113.7 (65.4, 181.4) | 8.8 (5.3, 14.5) |
| 54 | 2.4 (1.7, 3.1) | 99.2 (72.1, 132.3) | 10.1 (7.4, 13.6) |
| 55 | 0.7 (0.4,1.2) | 104.6 (60.2, 166.8) | 9.6 (5.8, 15.9) |
| 61 | 5.2 (4.2,6.3) | 94.7 (76.7, 115.4) | 10.6 (8.7, 12.9) |
| 71 | 0.6 (0.3,1.0) | 138.9 (81.5, 218.5) | 7.2 (4.4, 11.7) |
| 81 | 3.4 (2.6,4.3) | 111.1 (86.2, 140.3) | 9.0 (7.1, 11.5) |
| 83 | 0.7 (0.4,1.2) | 127.7 (73.5, 203.6) | 7.8 (4.7, 12.7) |
| 84 | 2.8 (2.1,3.6) | 117.0 (88.5, 151.0) | 8.5 (6.6, 11.0) |

Supplemental Table 3 Univariate and multivariate analyses of factors associated with incidence and clearance of any HPVs, hR-HPVs and Lr-HPVs according to HPV group

| **Determinant** | **Incidence** | | **Clearance** | |
| --- | --- | --- | --- | --- |
|  | **Univariate** | **Multivariate** | **Univariate** | **Multivariate** |
| **Any HPV** | HR(95%CI) | HR(95%CI) | HR(95%CI) | HR(95%CI) |
| **Age** |  |  |  |  |
| ≤34 years | ref |  | ref |  |
| ≥35 years | **1.33(1.05,1.68)** | 1.05 (0.71,1.55) | 0.90(0.71,1.15) | 0.71(0.48,1.07) |
| **Census register** |  |  |  |  |
| Urumchi | ref |  | ref |  |
| Other cities | 0.94(0.75,1.18) |  | 0.90(0.71,1.13) |  |
| **Nation** |  |  |  |  |
| Han | ref |  | ref |  |
| minority | **1.49(1.02,2.18)** | 0.86(0.51,1.44) | 0.76(0.50,1.14) | 0.46(0.27,0.77) |
| **Education** |  |  |  |  |
| Less than or equal to high school | ref |  | ref |  |
| Higher professional education or university | 0.82(0.61,1.10) |  | 1.10(0.82,1.48) |  |
| **Employment** |  |  |  |  |
| Employed | ref |  | ref |  |
| Unemployment/Unemployed | 1.37(0.94,1.99) |  | 1.44(0.98,2.12) |  |
| **Marital Status** |  |  |  |  |
| Married | ref |  | ref |  |
| Unmarried | **0.73(0.55,0.98)** | 0.70(0.44,1.13) | 1.09(0.81,1.46) | 0.87(0.54,1.40) |
| Divorced/widowed | 0.93(0.60,1.45) | 1.20(0.62,2.32) | 1.02 (0.65,1.61) | 1.43 (0.74,2.75) |
| **Monthly income** |  |  |  |  |
| ≤5000 RMB | ref |  | reef |  |
| >5000 RMB | 0.91(0.73,1.14) |  | 0.96(0.76,1.20) |  |
| **Sexual preference** |  |  |  |  |
| Homosexuality | ref |  | ref |  |
| Heterosexuality/others | 1.11(0.84,1.48) |  | 1.02(0.77,1.36) |  |
| **Gender of lifelong sexual partners** |  |  |  |  |
| Male only | ref |  | ref |  |
| Both male and female | 1.13(0.90,1.42) |  | 0.91(0.73,1.15) |  |
| **Gender of sexual partners in the past year** |  |  |  |  |
| Male only |  |  |  |  |
| Both male and female | 1.14(0.85,1.54) |  | 0.86 (0.64,1.15) |  |
| **Homosexual anal sex in the past 6 months** |  |  |  |  |
| YES | 0.87(0.66,1.14) |  | 0.76(0.58,1.01) |  |
| NO | ref |  | ref |  |
| **Anal sexual position** |  |  |  |  |
| Mainly inserted anal sex |  |  |  |  |
| both inserted and receptive | **1.64(1.17,2.29)** | **1.74(1.06,2.84)** | **0.93(0.67,1.30)** | 0.95(0.59,1.52) |
| Mainly receptive anal sex | **1.37(1.06,1.77)** | **1.66(1.16,2.38)** | **0.65(0.50,0.85)** | 0.66(0.46,0.95) |
| **Number of sexual partners past 6 months** |  |  |  |  |
| <5 |  |  |  |  |
| ≥5 | **1.52(1.00,2.30)** | 1.28(0.78,2.09) | 1.19(0.79,1.79) | 1.07(0.66,1.74) |
| **Latest anal sex condom use** |  |  |  |  |
| Yes | reg |  | reg |  |
| NO | **1.60(1.11,2.31)** | **1.80(1.10,2.94)** | 1.21(0.83,1.77) | 1.47(0.88,2.45) |
| **Condom use during anal sex in the past 6 months** |  |  |  |  |
| always with condoms |  |  |  |  |
| sometimes with condoms | 1.07(0.80,1.42) |  | 1.01(0.76,1.34) |  |
| never with condoms | 0.88(0.54,1.42) |  | 0.99(0.61,1.61) |  |
| **Number of anal in the past a week** |  |  |  |  |
| <2 | ref |  | ref |  |
| ≥2 | 0.91(0.68,1.22) |  | 0.89(0.67,1.19) |  |
| **Had commercial sex with men in the past 6 months** |  |  |  |  |
| No | ref |  | ref |  |
| Yes | 1.82(0.97,3.39) |  | 1.36 (0.71,2.60) |  |
| **Circumcision** |  |  |  |  |
| Yes | ref |  | ref |  |
| No | 0.88(0.70,1.12) | 0.91(0.65,1.27) | **0.74(0.58,0.94)** | 0.74(0.53,1.03) |
| **Had sex with heterosexual partners in the past 6 months** |  |  |  |  |
| No | ref |  | ref |  |
| Yes | 0.99(0.72,1.39) |  | 0.89(0.65,1.23) |  |
| **Age of sexual debut** |  |  |  |  |
| ≥18 | ref |  | ref |  |
| <18 | 1.18(0.84,1.67) |  | 1.05(0.75,1.48) |  |
| **Drug use past 6 months** |  |  |  |  |
| No | ref |  | ref |  |
| Yes | **1.252(0.98,1.61)** | **1.41(1.00,1.97)** | 1.09(0.85,1.40) | 1.15(0.82,1.61) |
| **Smoking** |  |  |  |  |
| Never smoked |  |  |  |  |
| Sometimes | 0.79(0.57,1.09) |  | 0.92(0.66,1.28) |  |
| Smoking every day | 0.83(0.63,1.09) |  | 1.01(0.77,1.33) |  |
| **Drinking** |  |  |  |  |
| Never drink |  |  |  |  |
| Sometimes | 1.00(0.79,1.26) |  | 1.17(0.93,1.49) |  |
| Drink every day | 0.59(0.30,1.18) |  | 0.70(0.31,1.57) |  |
| **History of STI** |  |  |  |  |
| No |  |  |  |  |
| Yes | **1.71(1.12,2.59)** | **2.17(1.10,4.26)** | 0.84(0.55,1.30) | 1.10(0.54,2.22) |

| **Determinant** | **Uninfected → Infected** | | **Infected→ Uninfected** | |
| --- | --- | --- | --- | --- |
|  | **Univariate** | **Multivariate** | **Univariate** | **Multivariate** |
| **Hr-HPV** | HR(95%CI) | HR(95%CI) | HR(95%CI) | HR(95%CI) |
| **Age** |  |  |  |  |
| ≤34 years | ref |  | ref |  |
| ≥35 years | **1.29(1.02,1.64)** | 1.14 (0.79,1.65) | 0.95(0.75,1.21) | 0.88(0.61,1.28) |
| **Census register** |  |  |  |  |
| Urumchi | ref |  | ref |  |
| Other cities | 0.94(0.74,1.18) |  | 0.99(0.79,1.25) |  |
| **Nation** |  |  |  |  |
| Han | ref |  | ref |  |
| minority | 1.30(0.89,1.90) |  | 0.88(0.59,1.31) |  |
| **Education** |  |  |  |  |
| Less than or equal to high school | ref |  | ref |  |
| Higher professional education or university | 0.77(0.57,1.03) |  | 1.01(0.75,1.36) |  |
| **Employment** |  |  |  |  |
| Employed | ref |  | ref |  |
| Unemployment/Unemployed | 1.04(0.71,1.52) |  | 1.31(0.89,1.93) |  |
| **Marital Status** |  |  |  |  |
| Married | **ref** |  | ref |  |
| Unmarried | **0.67(0.50,0.91)** | 0.68(0.44,1.03) | 0.94 (0.70,1.27) | 0.90 (0.58,1.38) |
| Divorced/widowed | 0.821(0.53,1.27) | 1.13(0.57,2.25) | 0.79 (0.51,1.24) | 1.27(0.63,2.52) |
| **Monthly income** |  |  |  |  |
| ≤5000 RMB | ref |  | reef |  |
| >5000 RMB | 0.83(0.66,1.05) |  | 0.81(0.65,1.02) |  |
| **Sexual preference** |  |  |  |  |
| Homosexuality | ref |  | ref |  |
| Heterosexuality/others | 1.02(0.77,1.36) |  | 1.06(0.79,1.41) |  |
| **Gender of lifelong sexual partners** |  |  |  |  |
| Male only | ref |  | ref |  |
| Both male and female | 1.08(0.86,1.36) |  | 1.02(0.81,1.28) |  |
| **Gender of sexual partners in the past year** |  |  |  |  |
| Male only |  |  |  |  |
| Both male and female | 1.33(0.98,1.80) |  | 1.17(0.86,1.58) |  |
| **Homosexual anal sex in the past 6 months** |  |  |  |  |
| YES | 0.84(0.64,1.11) |  | 0.80(0.60,1.06) |  |
| NO | ref |  | ref |  |
| **Anal sexual position** |  |  |  |  |
| Mainly inserted anal sex |  |  |  |  |
| both inserted and receptive | **1.39(1.01,1.92)** | 1.12(0.73,1.71) | **0.71(0.52,0.98)** | **0.60(0.40,0.89)** |
| Mainly receptive anal sex | **1.57(1.21,2.04)** | **1.99(1.39,2.85)** | **0.68(0.52,0.89)** | 0.71(0.50,1.02) |
| **Number of sexual partners past 6 months** |  |  |  |  |
| <5 |  |  |  |  |
| ≥5 | 1.14(0.76,1.72) |  | 1.08(0.74,1.59) |  |
| **Latest anal sex condom use** |  |  |  |  |
| Yes | reg |  | reg |  |
| NO | **2.02(1.38,2.96)** | **2.60(1.42,4.77)** | **1.53(1.03,2.28)** | **2.11 (1.12,3.98)** |
| **Condom use during anal sex in the past 6 months** |  |  |  |  |
| always with condoms |  |  |  |  |
| sometimes with condoms | 1.20(0.90,1.59) |  | 1.11(0.84,1.48) |  |
| never with condoms | 1.22(0.73,2.04) |  | 1.41(0.85,2.35) |  |
| **Number of anal in the past a week** |  |  |  |  |
| <2 | ref |  | ref |  |
| ≥2 | 0.79 (0.60,1.05) | 0.81(0.57,1.134) | **0.64(0.48,0.87)** | **0.61(0.43,0.87)** |
| **Had commercial sex with men in the past 6 months** |  |  |  |  |
| No | ref |  | ref |  |
| Yes | 1.86(0.93,3.72) |  | 1.65(0.81,3.36) |  |
| **Circumcision** |  |  |  |  |
| Yes | ref |  | ref |  |
| No | 1.09(0.86,1.39) |  | 0.81(0.64,1.02) |  |
| **Had sex with heterosexual partners in the past 6 months** |  |  |  |  |
| No | ref |  | ref |  |
| Yes | 1.08(0.77,1.53) |  | 1.15(0.83,1.60) |  |
| **Age of sexual debut** |  |  |  |  |
| ≥18 | ref |  | ref |  |
| <18 | 1.14(0.80,1.62) |  | 1.11(0.79,1.55) |  |
| **Drug use past 6 months** |  |  |  |  |
| No | ref |  | ref |  |
| Yes | 1.10(0.86,1.40) |  | 0.91(0.71,1.16) |  |
| **Smoking** |  |  |  |  |
| Never smoked |  |  |  |  |
| Sometimes | 0.71(0.51,1.00) | 0.98(0.61,1.58) | 0.86(0.61,1.20) | 1.23(0.77,1.95) |
| Smoking every day | **0.72 (0.54,0.94)** | 0.74(0.51,1.05) | 0.81(0.62,1.07) | 0.77(0.54,1.10) |
| **Drinking** |  |  |  |  |
| Never drink |  |  |  |  |
| Sometimes | 0.82(0.65,1.04) |  | 0.99(0.78,1.25) |  |
| Drink every day | 0.65(0.31,1.34) |  | 0.68(0.28,1.63) |  |
| **History of STI** |  |  |  |  |
| No |  |  |  |  |
| Yes | **1.51(1.02,2.22)** | 1.38(0.83,2.29) | 0.77(0.51,1.16) | 0.79(0.46,1.34) |

| **Determinant** | **Uninfected → Infected** | | **Infected→ Uninfected** | |
| --- | --- | --- | --- | --- |
|  | **Univariate** | **Multivariate** | **Univariate** | **Multivariate** |
| **Lr- HPV** | HR(95%CI) | HR(95%CI) | HR(95%CI) | HR(95%CI) |
| **Age** |  |  |  |  |
| ≤34 years | ref |  | ref |  |
| ≥35 years | **1.31(1.01,1.69)** | **1.40(1.02,1.93)** | 0.89(0.68,1.15) | 1.11(0.81,1.52) |
| **Census register** |  |  |  |  |
| Urumchi | ref |  | ref |  |
| Other cities | 0.97(0.76,1.25) |  | 0.82(0.64,1.06) |  |
| **Nation** |  |  |  |  |
| Han | ref |  | ref |  |
| minority | 1.55(1.06,2.26) |  | 0.76(0.51,1.11) |  |
| **Education** |  |  |  |  |
| Less than or equal to high school | ref |  | ref |  |
| Higher professional education or university | 1.00 (0.72,1.38) |  | 0.99(0.72,1.36) |  |
| **Employment** |  |  |  |  |
| Employed | ref |  | ref |  |
| Unemployment/Unemployed | 1.38(0.93,2.04) |  | 1.04(0.69,1.56) |  |
| **Marital Status** |  |  |  |  |
| Married | ref |  | ref |  |
| Unmarried | 0.93(0.68,1.26) |  | 1.34(0.99,1.81) |  |
| Divorced/widowed | 1.05(0.64,1.73) |  | 1.43(0.88,2.32) |  |
| **Monthly income** |  |  |  |  |
| ≤5000 RMB | ref |  | reef |  |
| >5000 RMB | 0.95(0.74,1.22) |  | 1.09(0.84,1.40) |  |
| **Sexual preference** |  |  |  |  |
| Homosexuality | ref |  | ref |  |
| Heterosexuality/others | 1.07(0.79,1.44) |  | 0.78(0.58,1.06) |  |
| **Gender of lifelong sexual partners** |  |  |  |  |
| Male only | ref |  | ref |  |
| Both male and female | 1.22(0.95,1.57) |  | 0.83(0.65,1.07) |  |
| **Gender of sexual partners in the past year** |  |  |  |  |
| Male only |  |  |  |  |
| Both male and female | 0.87(0.63,1.18) | 0.85(0.58,1.25) | **0.57(0.42,0.77)** | **0.56(0.39,0.82)** |
| **Homosexual anal sex in the past 6 months** |  |  |  |  |
| YES | 1.34(0.98,1.84) |  | 0.95(0.69,1.29) |  |
| NO | ref |  | ref |  |
| **Anal sexual position** |  |  |  |  |
| Mainly inserted anal sex |  |  |  |  |
| both inserted and receptive | 1.21(0.85,1.71) |  | 0.79(0.56,1.10) |  |
| Mainly receptive anal sex | 1.30(0.98,1.72) |  | 0.77(0.58,1.02) |  |
| **Number of sexual partners past 6 months** |  |  |  |  |
| <5 |  |  |  |  |
| ≥5 | **1.81(1.14,2.88)** | 1.45(0.89,2.37) | 1.29(0.82,2.02) | 1.25(0.79,1.99) |
| **Latest anal sex condom use** |  |  |  |  |
| Yes | reg |  | reg |  |
| NO | 1.01(0.70,1.44) |  | 0.94(0.65,1.36) |  |
| **Condom use during anal sex in the past 6 months** |  |  |  |  |
| always with condoms |  |  |  |  |
| sometimes with condoms | 0.86(0.63,1.18) |  | 0.90(0.67,1.22) |  |
| never with condoms | 1.15(0.64,2.04) |  | 1.15(0.63,2.11) |  |
| **Number of anal in the past a week** |  |  |  |  |
| <2 | ref |  | ref |  |
| ≥2 | 1.254(0.90,1.74) |  | 1.15(0.84,1.59) |  |
| **Had commercial sex with men in the past 6 months** |  |  |  |  |
| No | ref |  | ref |  |
| Yes | 1.46(0.77,2.76) |  | 0.94(0.49,1.81) |  |
| **Circumcision** |  |  |  |  |
| Yes | ref |  | ref |  |
| No | 0.96(0.74,1.24） |  | 0.91(0.70,1.17) |  |
| **Had sex with heterosexual partners in the past 6 months** |  |  |  |  |
| No | ref |  | ref |  |
| Yes | 1.10(0.78,1.54) |  | 0.68(0.48,0.96) |  |
| **Age of sexual debut** |  |  |  |  |
| ≥18 | ref |  | ref |  |
| <18 | 1.13(0.78,1.63) |  | 1.06(0.74,1.50) |  |
| **Drug use past 6 months** |  |  |  |  |
| No | ref |  | ref |  |
| Yes | **1.90(1.43,2.53)** | **2.18(1.57,3.02)** | **1.67(1.26,2.20)** | **1.75(1.28,2.41)** |
| **Smoking** |  |  |  |  |
| Never smoked |  |  |  |  |
| Sometimes | 0.95(0.65,1.37) | 0.71(0.44,1.14) | 1.09(0.75,1.57) | 1.25(0.80,1.94) |
| Smoking every day | 1.13(0.82,1.56) | 1.09(0.72,1.65) | **1.52 (1.11,2.09)** | 1.45(0.96,2.17) |
| **Drinking** |  |  |  |  |
| Never drink |  |  |  |  |
| Sometimes | **1.36 (1.05,1.77)** | 1.23(0.89,1.71) | **1.37(1.06,1.78)** | 1.18(0.85,1.62) |
| Drink every day | 0.511(0.20,1.28) | 0.37(0.12,1.18) | 1.07(0.48,2.34) | 0.68 (0.21,2.20) |
| **History of STI** |  |  |  |  |
| No |  |  |  |  |
| Yes | 1.35(0.90,2.02) |  | 0.79 (0.52,1.21) |  |
